# Experimental evaluation of silicon carbide P-N detectors under thermal and fast neutron irradiation at the RA-6 nuclear research reactor

**DOI:** 10.1038/s41598-025-32175-8

**Published:** 2025-12-19

**Authors:** Martín Pérez, Juan Jerónimo Blostein, Felipe Zamorano, Celeste Fleta, Julio Marín, Giulio Pellegrini, Consuelo Guardiola

**Affiliations:** 1 Institute of Microelectronics of Barcelona (IMB-CNM-CSIC), Cerdanyola del Vallès (Bellaterra), 08193 Spain; 2 Comisión Nacional de Energía Atómica (CNEA), Av. Bustillo 9500, San Carlos de Bariloche, Río Negro Argentina; 3 Consejo Nacional de Investigaciones Científicas y Técnicas (CONICET), Buenos Aires, Argentina; 4 Instituto Balseiro, Av. Bustillo 9500, San Carlos de Bariloche, Río Negro Argentina

**Keywords:** Silicon carbide, Thermal neutron detection, Fast neutron detection, Radiation hard detectors, Energy science and technology, Engineering, Materials science, Physics

## Abstract

Silicon carbide (SiC) detectors present key advantages for neutron detection in harsh radiation environments, including high radiation hardness, low leakage current, and excellent thermal stability. In this work, we report on the characterization of SiC P–N diodes manufactured at the Institute of Microelectronics of Barcelona (IMB-CNM-CSIC) for both thermal and fast neutron detection. For thermal neutron measurements, 50 µm and 100 µm SiC diodes coupled to a ^6^LiF conversion layer were tested at the RA-6 Nuclear Research Reactor, San Carlos de Bariloche, Argentina. The detectors exhibited a linear response with reactor power up to 500 kW, with no evidence of saturation or dead-time effects, and an intrinsic detection efficiency of (4.39±0.22)%. The detection efficiency was characterized as a function of the neutron incidence angle, showing a monotonic increase. For fast neutrons, 100 µm diodes coupled to polypropylene layers of varying thicknesses were characterized using an AmBe source. The results showed that the intrinsic detection efficiency increased with converter thickness, reaching a maximum value of (0.57±0.04)% for an 800 µm layer. PHITS Monte Carlo simulations reproduced the experimental data, validating the interpretation of the measured spectra. These results confirm the potential of SiC-based detectors for reliable monitoring of thermal and fast neutrons in environments with high gamma background, including radiotherapy, nuclear reactors, and others.

## Introduction

Neutron detection is essential in various fields such as radiotherapy, radiation safety, nuclear security, and nuclear engineering^1^. Conventional silicon-based detectors are widely used, although they present important limitations, since they suffer radiation damage that reduces their lifetime and stability in harsh radiation environments^2–4^.

Silicon carbide (SiC) semiconductor detectors have gained attention as an alternative to Si due to their superior resistance under high fluences of energetic particles^5,6^. Previous research has investigated the use of SiC Schottky diodes for neutron detection. Hodgson et al.^7^ reports the characterization of a neutron detector based on a SiC–EP structure, consisting of a 50 µm n-type 4H-SiC epitaxial layer with Ti/Pt/Au Schottky contacts. Coutinho et al.^8^ examines the performance of SiC-based neutron detectors, addressing challenges related to material properties, device fabrication, and testing procedures. Additionally, Giudice et al.^9^ describes the evaluation of large-area 4H-SiC Schottky diodes coupled with a ^6^LiF converter for applications in Boron Neutron Capture Therapy (BNCT). Despite these advances, P–N junction diodes are generally preferred over Schottky diodes for radiation detection. In the case of comparing SiC with Si, this preference arises because the lower barrier height of Schottky contacts typically leads to higher reverse leakage currents compared to P–N junctions, resulting in increased power consumption and electric noise. Nevertheless, the fabrication of high-quality SiC P–N junctions remains technologically demanding, and only a limited number of studies have investigated their response to thermal neutrons. Issa et al. reports the development of neutron detectors based on SiC P–N diodes incorporating boron conversion layers implanted within the device structure^10^. The test performed by Obraztsova et al.^11^ compares the performance of a LiF-coated SiC P–N diode with that of a diamond detector for the detection of thermal neutrons generated in a nuclear reactor; another interesting features about diamond detectors covered with LiF and their properties are addressed by Sussmannet al.^12^. In addition, References^13–15^ focus on the detection of fast neutrons with SiC devices by means of nuclear reactions of Si and C present in the detectors and the nuclear recoil of these materials.

In this context, our group assessed the performance of 4H-SiC detectors fabricated at the Institute of Microelectronics of Barcelona (IMB-CNM-CSIC) under both thermal and fast neutron irradiation. Pérez et al.^16^ reported a characterization carried out at the HiSPANoS facility of the National Center of Accelerators in Seville, Spain, where the detector response was evaluated using a non-collimated thermal neutron beam as well as quasi-monoenergetic fast neutrons. Subsequently, in Reference^17^, we have presented measurements of out-of-the-field thermal neutrons generated by a radiotherapy LINAC, and it was demonstrated that the detectors used have a very low gamma sensitivity.

This work aimed at developing a matrix of SiC detectors for the characterization of neutron fluxes in pulsed radiotherapy accelerators. The neutron spectra typically observed in LINAC radiotherapy accelerators consist of a fast-neutron component that undergoes moderation within the bunker, leading to the production of thermal and epithermal neutron components. For a more detailed discussion of typical neutron spectra, the reader is referred to Kry et al. and Zamorano et al.^18,19^. In this study, we present the characterization of two neutron detectors based on SiC P–N diodes. These detectors will serve for the construction of the future detector array for further neutron spectroscopy characterization in radiation-hard environments. For this reason, the characterization was carried out under conditions representative of those expected in radiotherapy accelerators, where a background of thermal neutrons is present, impinging on the detector from all directions. Therefore, in this work we evaluated the intrinsic detection efficiency of the device —defined as the probability that a neutron incident on the detector is detected— as a function of the incident angle of a thermal neutron beam. In addition, the performance of the detectors is investigated under different bias voltages and neutron fluxes. Second, it is known that the use of fast neutron detectors based on moderators can hinder the measurement of the time structure of pulsed fast neutron beams^20^. For this reason, in the present work we evaluate fast neutron detectors consisting of SiC diodes covered with polypropylene layers, where the neutrons are detected through recoil protons generated by hydrogen atoms. We also present a characterization performed with an AmBe fast neutron source, in which the detection efficiency was studied as a function of the polyethylene layer thicknesses, as well as the dependence of the detector response on the applied bias voltage.

## Materials and methods

### SiC neutron detectors

**Fig. 1:**
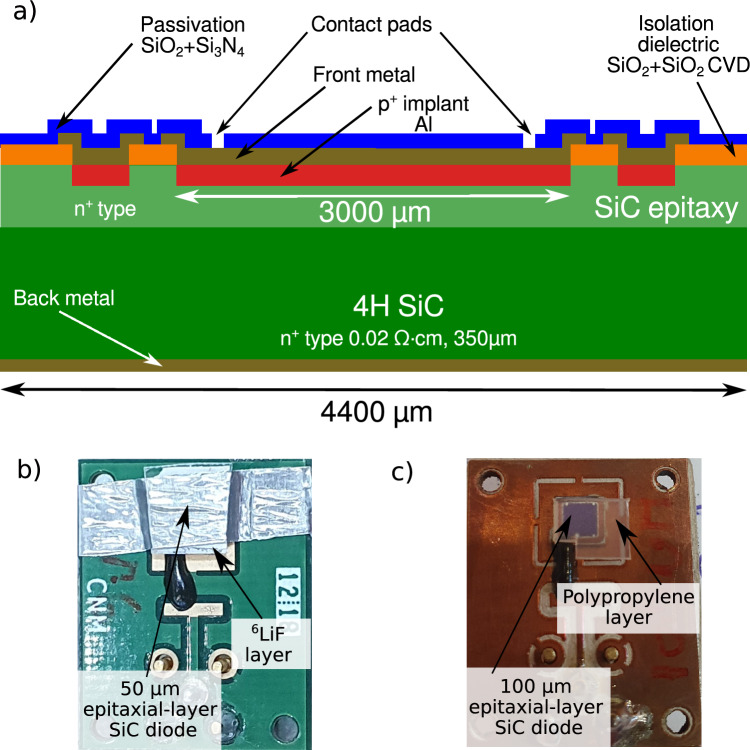
(**a**) Scheme of the detector cross-section. Lateral dimensions and thicknesses not to scale. (**b**) Thermal neutron detector: SiC diode covered with ^6^LiF. (**c**) Fast neutron detector: SiC Diode covered with polypropylene.

The measurements presented in this work were carried out using two P-N SiC diodes fabricated at IMB-CNM-CSIC. Figure 1a illustrates the detector cross-section. The devices were manufactured on 4H-SiC wafers and feature an active area of 3 × 3 $\hbox {mm}^2$. To form the P-N junction, a highly doped $\hbox {P}^+$ layer was created via Al ion implantation. A $\hbox {SiO}_2$/$\hbox {Si}_3\hbox {N}_4$ passivation layer protects the devices from environmental degradation. The front metal contacts are composed of a multilayer Ti/Al/Ti/Ni structure, while the back contact uses a Ti/Ni/Au multilayer^6,21^, the thickness of these layers is approximately 200 nm.

For thermal neutron detection, we employed a SiC diode with a 50 µm-thick epitaxial layer and a doping concentration of $1 \times 10^{14}$ $\hbox {cm}^{-3}$ (Fig. 1b), coupled to a ($50 \pm 10$) µm-thick ^6^LiF - 95% ^6^Li neutron converter fabricated following the method described in Perez *et al.* ^16,17^. In addition, a SiC diode with a 100 µm-thick epitaxial layer and a doping concentration of $4 \times 10^{14}$ $\hbox {cm}^{-3}$ was also tested in combination with the ^6^LiF converter for thermal neutron measurements. In the case of lithium, the isotope ^6^Li presents the largest neutron capture cross-section, and its interaction with thermal neutrons proceeds via the reaction:

1 $$\begin{aligned} \text {n}+ ^{6}\text {Li} \rightarrow \, ^{3}\text {H}\ (2.73\ \text {MeV}) + \alpha \ (2.05\ \text {MeV}), \end{aligned}$$

where a 2.73 MeV tritium nucleus and a 2.05 MeV alpha particle are emitted isotropically and in opposite directions in the laboratory reference system. The opposite directions of the emitted tritium nucleus and the alpha particle are due to linear momentum conservation; the target lithium nucleus is essentially at rest, and the total energy released in this nuclear reaction (Q = 4.78 MeV) is significantly greater than the kinetic energy of the incident neutron. For fast neutron detection, the same 100 µm device was used in combination with polypropylene converters of 300, 500, 800, and 1200 µm thickness (Fig. 1c).

### Readout electronics

The pulses generated by the detectors were amplified by means of a Cividec Cx-L charge-sensitive preamplifier with an integrated pulse shaper, and then digitized with open-source software implemented on a RedPitaya STEMlab 125-14 board^22,23^. The electronics were powered by batteries and a voltage regulator to minimize noise from the power line. We used a lower level discriminator (LLD) of 250 keV for all detectors.

### Energy calibration

For energy calibration purposes, the sensors were exposed to alpha particles emitted by a triple-peaked calibration source containing $^{239}$Pu, $^{241}$Am, and $^{244}$Cm. These isotopes emit alpha particles with energies of 5.15 MeV, 5.48 MeV, and 5.9 MeV, respectively. To perform the calibration we follow the procedure presented in References^16,17^. The source was placed 12 mm away from the surface of the sensor chips. Under these conditions, the alpha particles lose energy as they travel through air and the sensors insulating layers, resulting in maximum deposited energies of 4.0 MeV, 4.4 MeV, and 4.9 MeV, as determined through simulations performed with PHITS^24^.

### Thermal neutron irradiation

**Fig. 2:**
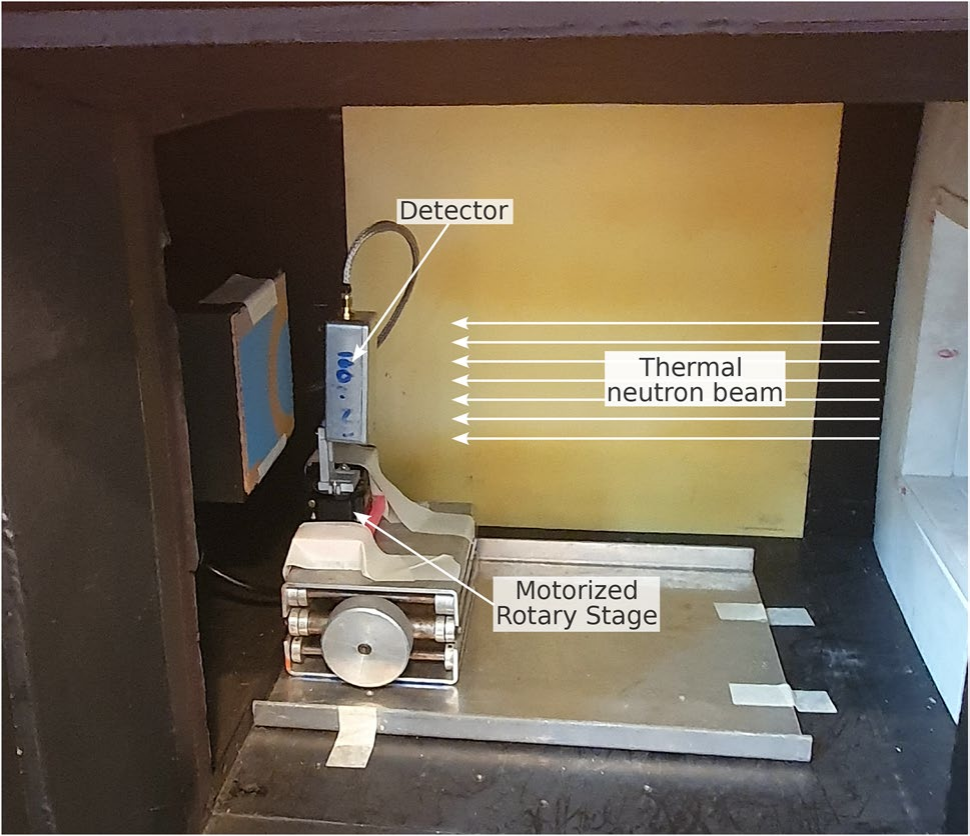
Experimental setup used in the experiments with thermal neutrons. Detector mounted over a motorized rotary stage inside the STORNI neutron imaging facility enclosure.

**Fig. 3:**
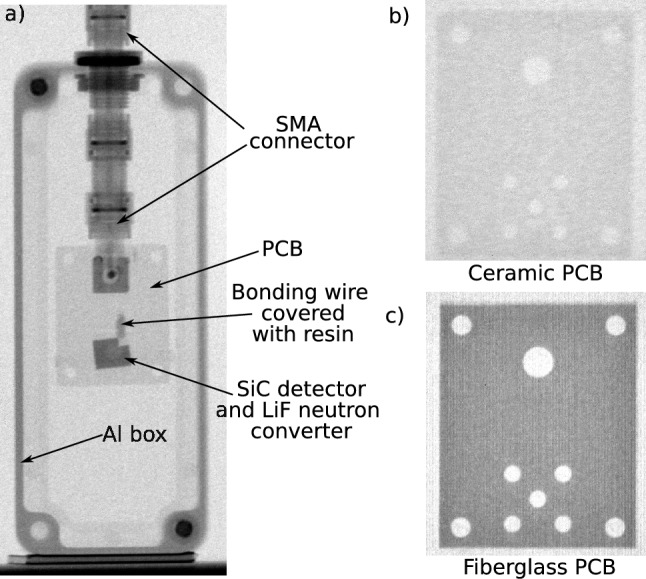
(**a**) Neutron radiography of the detector mounted on a ceramic PCB in an aluminum box using the standard STORNI facility. Neutron radiograph of a ceramic PCB (**b**), and fiberglass PCB (**c**).

The sensors were exposed to a neutron beam, dedicated to the STORNI neutron imaging facility of the RA-6 Nuclear Research Reactor^25^. It consists of collimated thermal and epithermal neutrons, along with gamma radiation in a wide energy spectrum from 0.1 to 10.5 MeV typical from the $^{235}$U fission fragments. At the detector position, for the employed irradiation configuration, the gamma dose is approximately 1 Sv/h. The facility is housed within a shielded enclosure designed for sample irradiation. Figure 2 shows the experimental setup used for the irradiations. The sensors were mounted on a motorized stage that was used to rotate the detectors with respect to the neutron beam. Figure 3a shows a neutron radiography of the detector mounted on a ceramic printed circuit board (PCB) in an aluminum box using the standard STORNI facility. The 50 µm detector, was mounted on a standard fiberglass PCB which among other components contains boron oxide $\hbox {B}_2\hbox {O}_3$, while the 100 µm detector was mounted on a ceramic board. Figure 3b,c show a comparison of the neutron radiographs of both PCB. It is worth to notice that the neutron attenuation in the ceramic PCB is lower than the one of the fiberglass PCB.

### Fast neutron irradiations

**Fig. 4:**
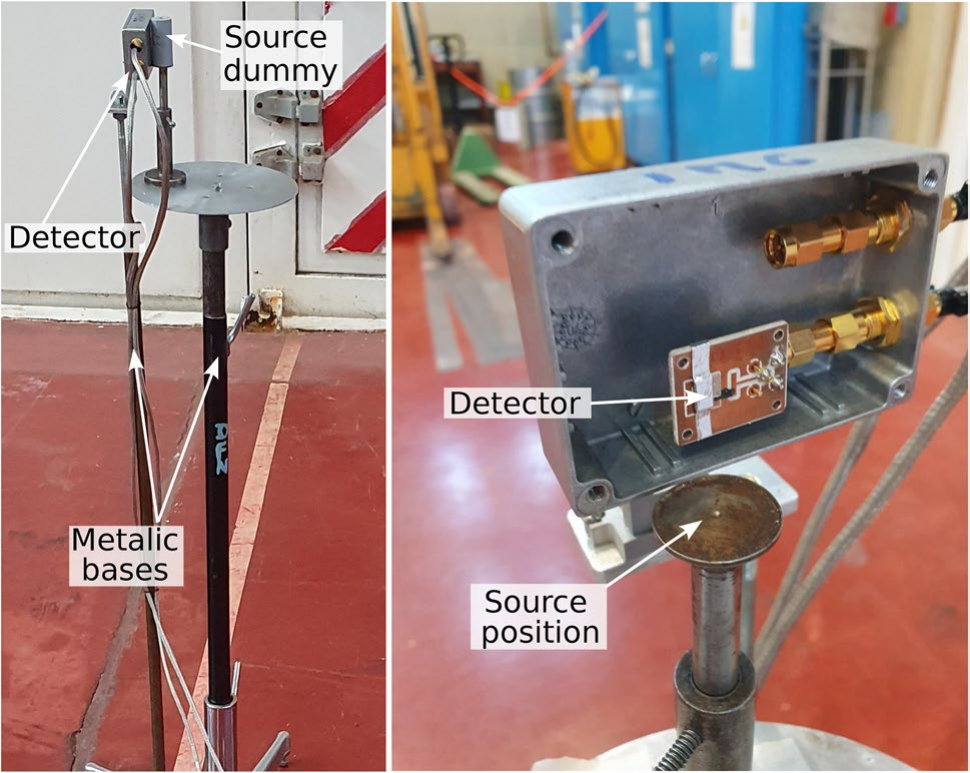
Experimental setup employed for the fast neutrons irradiation. The detector and the AmBe source were placed over metallic bases, the distance between the source and the detector was (12±1) mm.

Fast neutron experiments were performed in the RA-6 hall using an AmBe source with an activity of (103±5) GBq and a total isotropic neutron production of 6.9(0.3)$\times {10}^6$ n$\cdot$ $\hbox {s}^{-1}$. During this fast neutron irradiation, the reactor was shut down. The measurements were taken in the reactor hall because it is a radiologically controlled area with controlled access and authorized for working with this type of radioactive source. Figure 4 shows the experimental setup used for these irradiations. The detectors, as well as the neutron source, were placed in metallic bases at more than 4 meters from the nearest wall to minimize the contribution of wall-scattered neutrons. The distance between the detector and the source was (12±1) mm.

### PHITS simulations

The detector response was modeled using the PHITS simulation code, which is based on the Monte Carlo (MC) method. PHITS was developed through a collaboration between the Japan Atomic Energy Agency (JAEA) and various international research institutions^24^. In this study, version 3.34 of PHITS was used, together with the updated JENDL-5.0 nuclear data library. The simulation replicated the geometry of the experimental setup, including the aluminum housing, PCB, plastic support, diode characteristics, and neutron conversion layers. The energy deposition in the detector was calculated using the [T-Deposit] tally, which provides the spatial distribution of the deposited energy. For simulations involving fast neutrons, we employed the neutron spectrum emitted by an AmBe source. In the case of thermal neutron simulations, we employed a monochromatic neutron beam with an energy of 25 meV.

## Results and discussion

### Thermal neutron irradiations

**Fig. 5:**
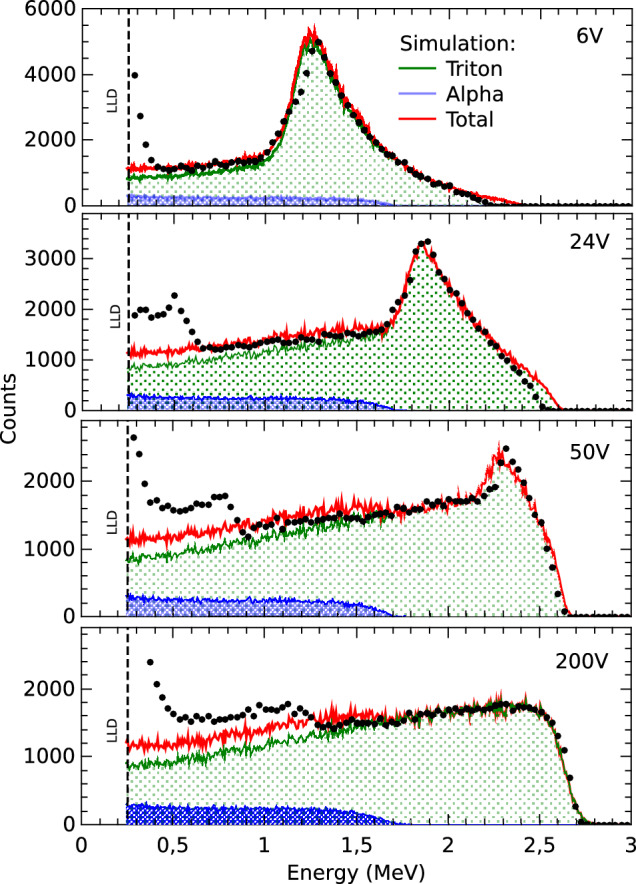
Pulse height spectra obtained during thermal neutron irradiations as a function of the polarization voltage. Black dots: experimental data. Red curves: result of the simulated spectra. Blue zones: contribution of alpha particles. Green zones: contribution of triton ions.

Firstly, the detector response is studied as a function of the applied bias voltage, to evaluate its dependence on the effective depletion depth. This approach enables the investigation of how the active thickness of the detector influences its response. Figure 5 presents the response of the 50 µm detector for 6, 24, 50, and 200 V. The dots correspond to the measurements, and the red curves show the results of the simulations performed with PHITS. In addition, the shaded areas correspond to the contributions of alpha particles (blue) and tritium ions (green) generated in the conversion layer. As shown in McGregor et al.^26^ the average range for the 2.73 MeV triton in ^6^LiF is 32.1 µm, while the average range for the 2.05  MeV alpha particles in this material is 6.11 µm. Table 1 presents a comparison of the ranges of the alpha particles and triton ions in LiF, SiC, and the thicknesses of the depletion region as a function of the bias voltage.

When bias voltages between 6 and 50 V are applied, a peak associated with tritium ions can be observed. This peak appears because the range of these ions in SiC (approximately 27 µm) exceeds the depletion depth at those voltages: 5, 12, and 18 µm for 6, 24, and 50 V, respectively. Consequently, a significant number of events generated by ions emitted perpendicularly to the detector surface, or at small angles relative to it, deposit part of their kinetic energy within an almost constant SiC thickness. These ions leave the depletion zone before reaching the Bragg peak, thus producing the observed peak in the pulse-height spectra. Since ion emission is isotropic, a fraction of ions travels nearly parallel to the detector surface. These ions move entirely within the depleted region, regardless of its thickness, generating a tail in the spectrum that extends up to 2.7 MeV, corresponding to the full energy of the nuclear reaction. As the bias voltage increases, the peak shifts toward higher energies because the region where tritium ions deposit their charge becomes thicker. At 200 V, the depletion depth reaches approximately 34 µm, allowing tritium ions to be completely stopped within this region. In this case, the pulse height no longer depends on the depletion width but is determined solely by the energy deposited by the ions. This energy is not unique but rather distributed almost uniformly from 0 to 2.7 MeV, due to variations in interaction depth and emission angle within the ^6^LiF layer. As a result, the spectrum exhibits a plateau with a cut-off near the upper limit of 2.7 MeV. This results are consistent with that reported by Giudice et al., where silicon carbide Schottky diodes coated with LiF were used for the measurement of thermal neutrons^9^. It can also be observed that the signal component originating from alpha particles remains practically constant for all bias voltages. This behavior is explained by the fact that their range in SiC (4.9 µm) is smaller than the depletion depth for all the voltages used. At low energies, the experimental curves exhibit a higher number of events compared to the simulations. This behavior arises from an edge effect of the detector. This was confirmed during an alpha particle irradiation, in which the detectors were covered with a mask that exposed only the central area; under these conditions, the effect was not observed. The edge effect can be mainly attributed to the diffusion of charge carriers generated less than one diffusion length outside the diode area, which reach the depletion region and are collected by the electric field^27^. As a result, an excess of low-energy counts is observed, which is not reproduced in the PHITS simulations.

**Table 1:** Ranges in ^6^LiF, and SiC of alpha particles and triton ions produced by the ^6^Li reaction, together with the depletion region thicknesses as a function of the applied bias voltage.

Ranges in ^6^LiF (µm)	
Alpha	Triton
6.1	32.1
Ranges in SiC (µm)	
Alpha	Triton
4.9±0.3	27.5±0.5
Voltage (V)	Depletion depth (µm)
6	5.0±0.5
24	12±1
50	18±2
200	34±3

**Fig. 6:**
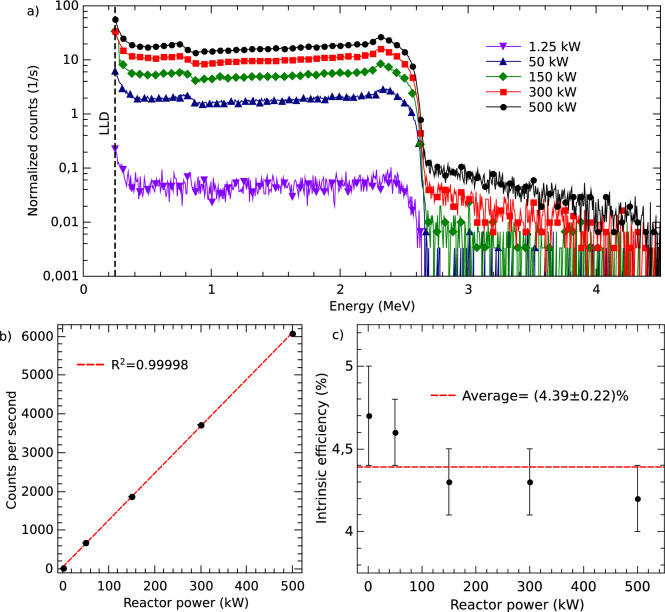
(**a**) Pulse height spectra obtained during thermal neutron irradiations as a function of reactor power, for a bias voltage of 50 V. (**b**) Count rate as a function of the reactor power. The statistical uncertainties are smaller than the symbol size. (**c**) Intrinsic detection efficiency as a function of the reactor power.

Secondly, we investigate the linearity as a function of reactor power. Figure 6a shows the pulse height spectra obtained with the 50 µm detector biased at 50 V for different power levels. The reactor was operated at 1.25, 50, 150, 300, and 500 kW. The spectra were obtained from measurements that lasted 5 minutes. Figure 6b shows the count rate as a function of the reactor power. The red curve corresponds to a linear fit of the experimental data. It is possible to observe that the detector response is linear within the investigated power range, and there is no evidence of saturation or dead-time effects.

At 500 kW the thermal neutron flux at the sensor position was 1.6(0.1)$\times {10}^6$ n$\cdot \hbox {cm}^{-2}\cdot \hbox {s}^{-1}$, this value was determined by standard neutron activation analysis with bare and cadmium-covered Mn foils. Figure 6c shows a plot of the intrinsic thermal neutron efficiency as a function of the reactor power. The red dashed line in the figure represents the weighted average intrinsic detection efficiency, which under these conditions was (4.39±0.22)%. This intrinsic efficiency is consistent with the theoretical value presented by McGregor et al.^26^ and in previous measurements^17^.

**Fig. 7:**
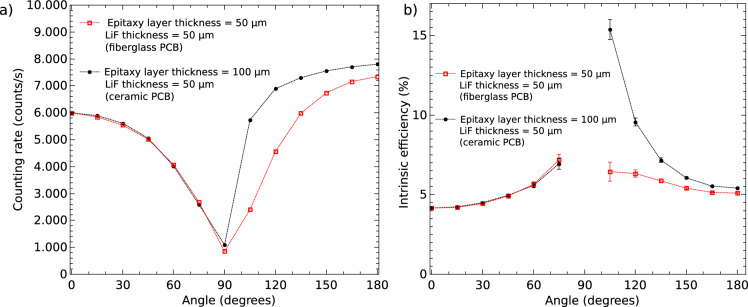
Counting rate (**a**), and intrinsic thermal neutron detection efficiency (**b**) as a function of the neutron incident angle.

To determine the intrinsic detection efficiency as a function of the neutron incidence angle, the detectors were mounted on a motorized stage and irradiated at different orientations. The measurements were performed at a reactor power of 500 kW, the maximum available, to maximize the counting rate thus reducing statistical uncertainty. The incidence angle was varied from $0^\circ$ (detector surface perpendicular to the neutron beam direction) to $180^\circ$, in steps of $15^\circ$. Figure 7a shows the intrinsic detection efficiency (IE) —defined as the ratio of detected neutrons to the number of neutrons incident on the detector— as a function of the incident angle. The measured quantity during a given acquisition time corresponds to the number of recorded events. Figure 7a shows the obtained counting rate as a function of the incident angle. Different acquisition times were selected to ensure comparable statistics for the various angles. In Fig. 7b we present the obtained intrinsic detection efficiency (IE) –defined as the ratio of detected neutrons to the number of neutrons incident on the detector– as a function of the incident angle. The IE was calculated as the counting rate (counts per second) over the neutron beam area exposed to the detector (in $\hbox {cm}^2$), and by the incident neutron flux (in neutrons per $\hbox {cm}^2$ per second). At $0^\circ$, the IE matches the value shown in Fig. 6c. As was explained by McGregor et al.^26^, when the incident angle increases, the IE rises because the effective thickness of the conversion layer increases, while the range of the secondary particles emitted in the layer remains constant. The results obtained at angles around $90^\circ$ were not included in Fig. 7b because they do not represent the intrinsic detection efficiency at such angles. This is so because the area of the conversion layer we have used is significantly larger than the area of the semiconductor depletion zone, and therefore, the ions produced by neutrons absorbed at the edge of the conversion layer cannot reach the depletion zone. Furthermore, the employed beam is not perfectly collimated; as can be seen in reference^25^, the beam used has been designed to have an angular divergence of L/D=100. For this reason, even if the detector were rotated exactly $90^\circ$, a fraction of neutrons would be incident on the front face of the detector, producing counts due to absorptions that do not occur at the edges of the conversion layer. Furthermore, this effect is increased if the detector is not orientated exactly at $90^\circ$, which is very difficult to guarantee. For incident angles greater than $90^\circ$, the IE decreases, reaching a value of about 5% for both detectors. At $180^\circ$ (back irradiation), the IE is higher than that at $0^\circ$, which is consistent with the results reported in Reference^26^. For this back-illuminated condition, as the thickness of the conversion layer increases, the subsequent parts of the conversion layer can only contribute to increasing the signal intensity. For angles above $90^\circ$, the 50 µm detector mounted on a standard fiberglass PCB exhibits a systematically lower efficiency than that of 100 µm device (mounted on a ceramic board). This reduction is due to the higher neutron absorption caused by the boron present in the standard PCB material, as can be observed in Fig. 3.

### Fast neutron irradiations

**Fig. 8:**
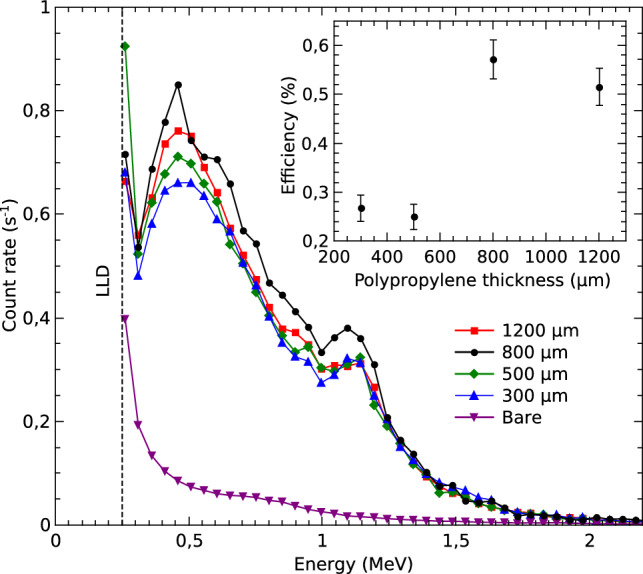
Spectra obtained during fast neutron irradiations for different polypropylene converter thicknesses, with the detector biased at 50 V.

In this section we will present the results of the irradiations with fast neutrons produced by the AmBe source. Figure 8 shows the spectra obtained with the 100 µm detector covered with 300, 500, 800, and 1200 µm of polypropylene, and with the bare detector. In these measurements the detector was biased at 50 V, resulting in an active thickness of 18 µm. The spectra were normalized to the live time, so the vertical axis represents the count rate of the measurements. In the case of the bare detector, the count rate is significantly lower than that produced in the detectors covered with polypropylene. The recorded events in the bare detector are mostly produced by the recoil of fast neutrons on C and Si nuclei within the detector. In contrast, in the covered detectors, most of the recorded events are due to protons generated by neutron recoil on the hydrogen atoms present in the polypropylene layers.

In the experimental conditions used, the fast neutron flux incident on the detector is (3.4±0.2)$\times {10}^4\hbox {ns}^{-1}$ as estimated from the AmBe source activity and the source–detector distance of (12.0±0.5) mm. The inset of Fig. 8 presents the intrinsic detection efficiency as a function of the polypropylene thickness; it is possible to observe that the efficiency ranges from 0.25 % to 0.57 % for thicknesses of 500 and 800 µm respectively.

**Fig. 9:**
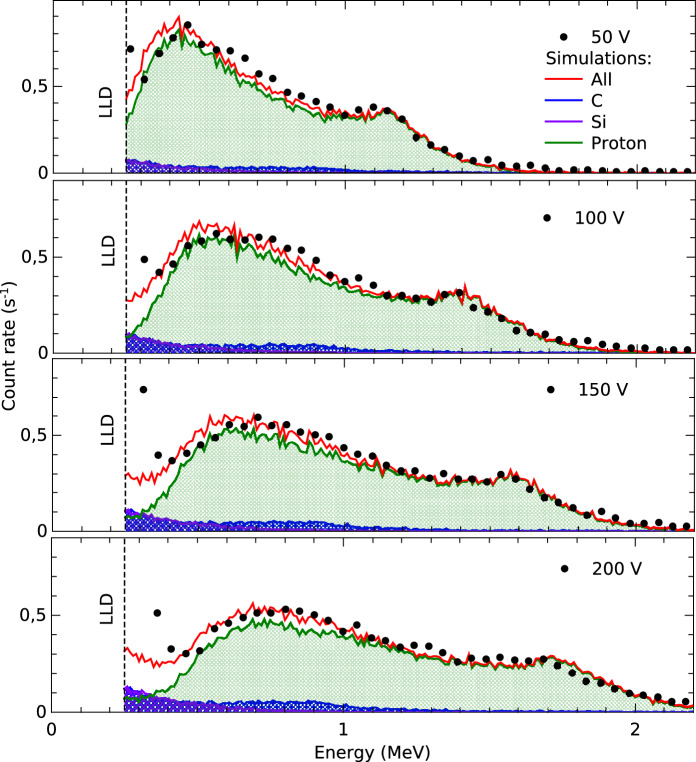
Response of the SiC diode covered with 800 µm of polypropylene for different bias voltages.

Figure 9 shows the response of the SiC diode covered with 800 µm of polypropylene for different bias voltages (black dots). The plots also include the results of the PHITS simulations: the red lines represent the total number of events, while the green and blue shaded areas correspond to the contributions from protons and carbon ions, respectively. As mentioned before, most of the recorded events are produced by recoil protons. The contribution of carbon ions is negligible, and the events produced by the recoil of Si ions deposit an amount of energy below the detection threshold of 250 keV. It can be observed that the charge deposited by protons increases with the applied bias voltage because of the increase in the thickness of the depletion region.

In this case, peaks similar to those described in Fig. 5 can also be observed. These peaks are produced by protons generated in the conversion layer whose range exceeds the depletion depth; they traverse the detector and deposit energy within a constant thickness of SiC, thus giving rise to a peak. The amplitude of these peaks decreases as the bias voltage increases and the depletion region becomes thicker, in the same way as observed for tritium ions produced by thermal neutrons.

## Conclusion

In this work, we experimentally characterized SiC P–N diodes for both thermal and fast neutron detection, supported by PHITS Monte Carlo simulations. For thermal neutrons, a SiC sensor coated with a 95% ^6^Li-enriched LiF conversion layer was employed. Measurements performed with a collimated thermal and epithermal neutron beam demonstrated that the detector pulse-height spectra at various bias voltages are consistent with the simulated response, with the signal predominantly generated by ionization from triton ions. The measured intrinsic thermal neutron detection efficiency, approximately 4.4% for normal incidence, is in agreement with values reported in the literature. In addition, the counting rate exhibited a linear dependence on reactor power, indicating negligible pile-up or dead-time effects up to about 6000 counts per second. The angular dependence of the intrinsic efficiency showed an increasing trend for grazing incidence, attributable to the increase in the effective thickness of the conversion layer, while the range of the secondary charged particles remains unchanged. For fast neutrons from an AmBe source, the SiC diodes combined with polypropylene conversion layers exhibit a maximum efficiency at a thickness of 800 µm. The detector response was found to depend on the applied bias voltage, and the experimental results showed good agreement with the simulations, confirming the detection mechanism based on recoil protons. Overall, the results demonstrate the robustness, stability, and effectiveness of SiC-based detectors and support their suitability for use in compact arrays for neutron monitoring in pulsed radiotherapy accelerators and other radiation-intensive environments.

## Data Availability

All data and the simulations of this work are available. There will not be restrictions to use the generated data after its publication, but external users will be asked to cite the source and/or the papers properly, and the redistribution of the data must be authorized by the corresponding author. The identity of the person accessing the data will be ascertained with a questionnaire, available at http://hdl.handle.net/10261/410361.
